# Clinical and molecular analysis of four unrelated Chinese families with pathogenic *KLHL40* variants causing nemaline myopathy 8

**DOI:** 10.1186/s13023-022-02306-9

**Published:** 2022-04-04

**Authors:** Haiming Yuan, Qingming Wang, Xiumei Zeng, Peiqing He, Wanfang Xu, Hongmei Guo, Yanhui Liu, Yangyang Lin

**Affiliations:** 1 Affiliated Dongguan Maternal and Child Health Care Hospital, Southern Medical University, Dongguan, 523120 China; 2 Dongguan Institute of Reproductive and Genetic Research, Dongguan, 523120 China

**Keywords:** *KLHL40*, Nemaline myopathy, Polyhydramnios, Fetal akinesia, Respiratory failure, Carrier screening

## Abstract

**Background:**

Homozygous or compound heterozygous variants in the *KLHL40* gene cause nemaline myopathy 8 (NEM8), a severe autosomal recessive muscle disorder characterized by prenatal polyhydramnios, fetal akinesia or hypokinesia, joint contractures, fractures, respiratory failure and dysphagia. Currently, 46 individuals with NEM8 have been described in the literature, and 30 variants in *KLHL40* have been identified.

**Results:**

Here, we reported five individuals from four unrelated Chinese families who presented common features of nemaline myopathy and infrequent clinical characteristics. Whole-exome sequencing (WES) was used to identify the causative gene. WES identified a recurrent missense variant c.1516A>C (p.Thr506Pro) and a novel frameshift variant c.543del (p.Ser182Profs*17) in *KLHL40* in patient 1, a nonsense variant c.602G>A (p.Trp201*) and a missense variant c.1516A>C (p.Thr506Pro) in *KLHL40* in patient 2, and homozygous variant c.1516A>C (p.Thr506Pro) in *KLHL40* in patient 3 and both siblings (patients 4 and 5), all of which were confirmed by Sanger sequencing. Next, we estimated the incidence of this disorder in the southern and northern Chinese population to be 4.59/10^6^ and 2.95/10^6^, respectively, based on the cumulative allele frequency of pathogenic variants in internal database.

**Conclusion:**

The results of our study expand the mutation spectrum of *KLHL40* and enrich our understanding of the clinical characteristics of NEM8. Genetic counseling was provided for the four families involved in this study. Given the severity and the relatively high incidence of this condition, we strongly suggest that *KLHL40* be incorporated into a carrier screening panel for the Chinese population.

**Supplementary Information:**

The online version contains supplementary material available at 10.1186/s13023-022-02306-9.

## Introduction

Nemaline myopathy (NEM) is a heterogeneous group of congenital myopathies with broad clinical phenotypes that range from mild muscle dysfunction to severe neonatal muscle weakness, often leading to fetal or early death. Currently, at least twelve genes have been recognized as causing nemaline myopathy. Some exhibit an autosomal recessive pattern, such as *CFL2, KLHL40, KLHL41, LMOD3, MYPN, NEB, TNNT1*. Less often, they exhibit an autosomal dominant pattern, including *KBTBD13, TNNT3* and *TPM2*, or an autosomal dominant/recessive pattern, such as *ACTA1,* TPM3 [1]. Nemaline myopathy 8 (MIM #615348), caused by biallelic pathogenic variants in *KLHL40*, is one of the most severe forms of NEM. It is characterized by early-onset severe generalized muscle weakness or hypokinesia, multiple joint contractures, fractures, respiratory failure and dysphagia apparent at birth. The average age of death is 5 months [2]. NEM8 is considered to be a very rare autosomal recessive muscle disorder, with only 46 NEM8 individuals being reported worldwide; 30 variants in *KLHL40* have been identified to date [2–9]. Recently, two genetic testing centers from southern China analyzed the frequency of pathogenic variants of *KLHL40* in their in-house database and proposed that the condition was not-so-rare in southern Chinese individuals, suggesting that the gene/variants should be included in a carrier screening panel and considered in prenatal diagnosis when congenital myopathies are suspected [8, 9]. However, this evidence is from only two centers and is limited. Our genetic testing center, also located in southern China, identified an additional five individuals with NEM8 from four unrelated Chinese families. The patients presented common features of NEM8, and the clinical features were delineated in detail. The results of our study expand the genetic variant spectrum of *KLHL40* and enrich our understanding of the clinical characteristics of this disorder, which will be beneficial for improving the prenatal diagnosis of NEM8. Furthermore, we provide further evidence that NEM8 has been historically underdiagnosed and that *KLHL40* should be listed in the carrier screening panel.

## Materials and methods

### Ethical compliance

This study was approved by the Ethics Committee of Dongguan Maternal and Child Health Care Hospital. Written informed consent was obtained from the legal guardians for the publication of any potentially identifiable images or data included in this article.

### Trio-based whole exome sequencing (WES)

Trio-based whole-exome sequencing was performed for families to screen for causal variants. Sequencing was performed with an Illumina NovaSeq 6000 (Illumina, San Diego, CA, USA). Suspected variants were verified by Sanger sequencing. The pathogenicity of the sequence variants was interpreted according to the American College of Medical Genetics and Genomics/Association for Molecular Pathology (ACMG/AMP) guidelines [10].

## Results

### Patient 1

**Fig. 1:**
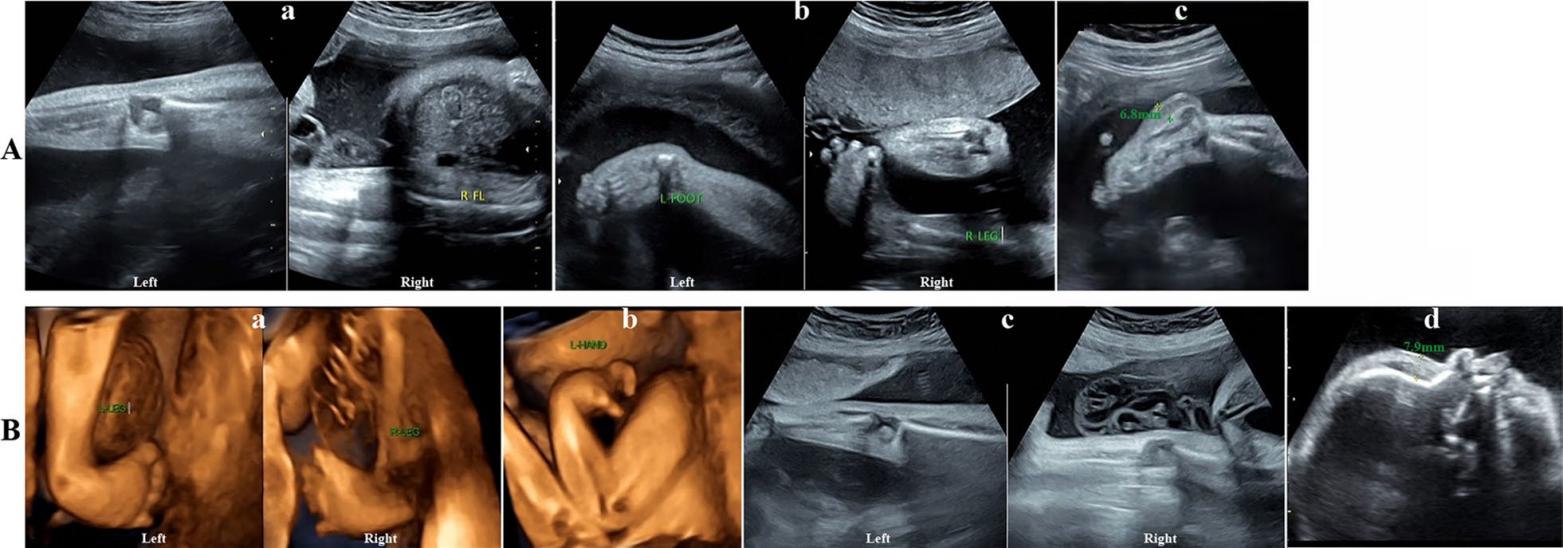
Ultrasound images. **A** Ultrasound images showing bilateral knee joint contractures (**a**), talipes equinovarus and inflexion (**b**), and increased thickness of planta skin (6.8 mm) (**c**) in patient 1. **B** Ultrasound images showing closed hands, flexed wrists (**a**), bilateral talipes equinovarus (**b**), extended legs and knee joint contractures (**c**), and thickening of the forehead soft tissue (7.9 mm) (**d**) in patient 3

A 30-year-old woman (gravida 2, para 1) felt reduced fetal movement compared with her previous pregnancy. The prenatal ultrasound scan showed obvious bilateral knee joint contractures, talipes equinovarus and inflexion and increased thickness of planta skin (6.8 mm) at 31 weeks (Fig. 1A). Karyotype and chromosomal microarray of amniocytes were normal. MLPA detected no deletion of exons 7 and 8 in *SMN1.* The women underwent vaginal delivery at 37 weeks. A female baby weighing 2250 g was born cyanotic and with dyspnea. The baby had generalized hypotonia, lack of spontaneous limb movements, bilateral elbow, hip and knee flexion contractures and mild skin edema. The baby passed away at 16 days of age due to severe respiratory failure and pneumonia.

**Fig. 2:**
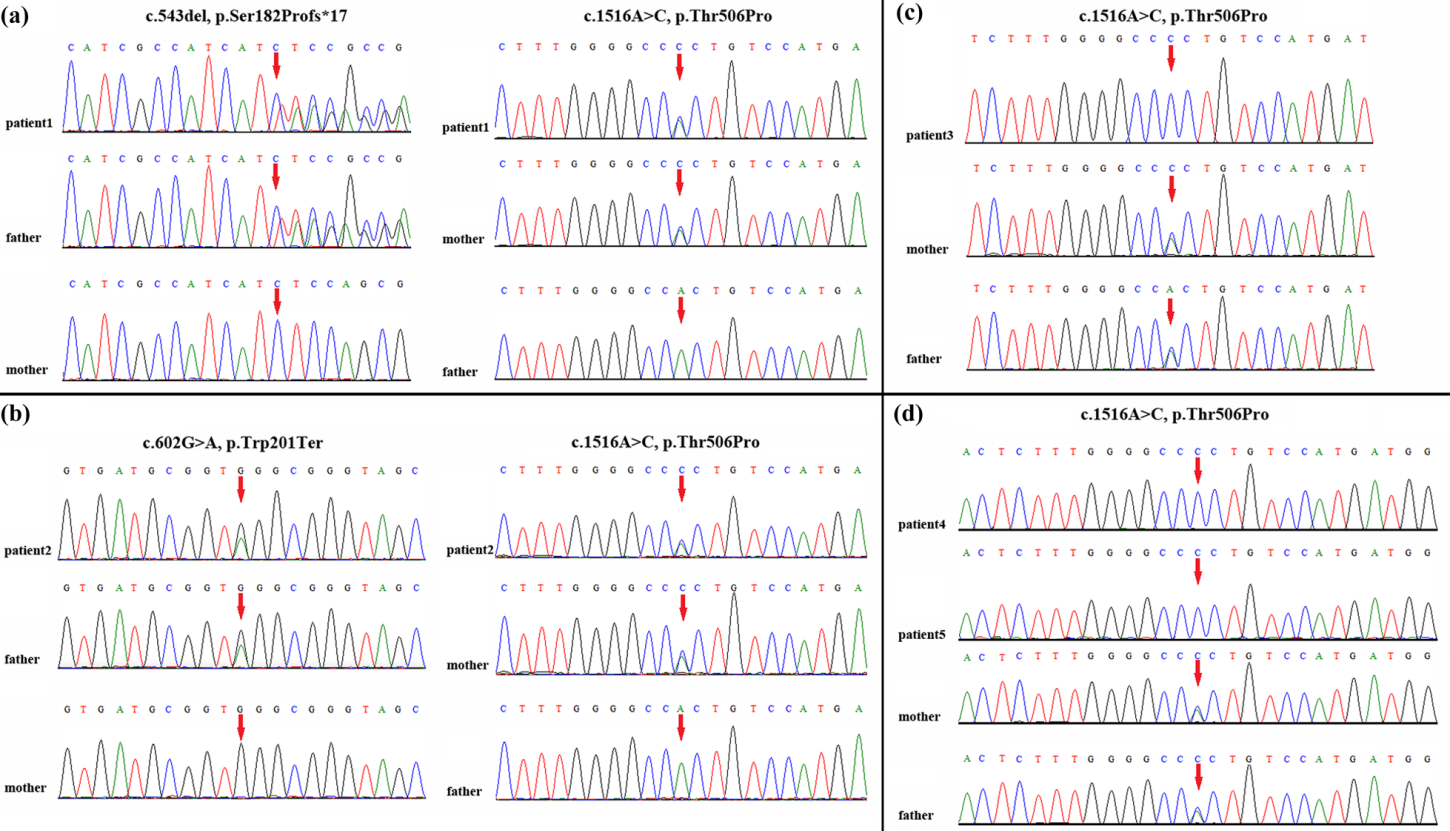
Variant identification by Sanger sequencing. Compound heterozygous variants c.543del and c.1516A>C in *KLHL40* were detected in patient 1 (**a**); compound heterozygous variants c.602G>A and c.1516A>C in *KLHL40* were detected in patient 2 (**b**); and homozygous variant c.1516A>C in *KLHL40* was detected in patient 3 (**c**) and both siblings (patients 4 and 5) (**d**). The red arrow indicates the variant site

Trio-based WES identified compound heterozygous variants, c.543del (p.Ser182Profs*17) and c.1516A>C (p.Thr506Pro) in *KLHL40* in the patient (Fig. 2a). Both asymptomatic parents were heterozygous carriers. The paternally inherited frameshift variant is novel, and it leads to a premature termination codon in the BACK domain that consequently results in loss-of-function of *KLHL40*. The maternally inherited missense variant has been proven to be a founder mutation in ethnic Chinese individuals [8]. Both variants can be classified as pathogenic according to the ACMG/AMP guidelines [10].

### Patient 2

A 28-year-old nulliparous woman presented with reduced fetal movement and progressive polyhydramnios. Amniocentesis showed a normal karyotype and chromosomal microarray. She underwent cesarean section at 35 weeks due to membrane rupture, and 2600 ml of liquid was noted during delivery. A male baby weighing 2150 g was born apneic and hypotonic. Multiple abnormalities were noted, including distinctive facial features (prominent forehead, hypertelorism, low-set ears and broad nasal bridge), severe hypotonia, multiple joint contractures, spontaneous limb fractures, bilateral clubfeet, talipes valgus, absent palmar and plantar crease, wide space between fingers and toes and cryptorchidism. The baby suffered from severe pneumonia and passed away at 20 days of age.

Trio-based WES identified compound heterozygous variants, c.602G>A (p.Trp201Ter) and c.1516A>C (p.Thr506Pro) in *KLHL40* in the patient, which were inherited from the father and mother, respectively (Fig. 2b). The paternally inherited nonsense variant was predicted to truncate the BACK domain of *KLHL40*. Both variants can be categorized as pathogenic according to the ACMG/AMP guidelines [10].

### Patient 3

A 27-year-old woman in her first pregnancy underwent an anomaly scan at 17 weeks of gestation, and the fetus was incidentally found to have bilateral talipes equinovarus. Persistent ultrasonic scans confirmed the condition at 21, 23 and 25 weeks of gestation. Then, the woman felt progressively reduced fetal movement. Amniocentesis showed a normal karyotype and chromosomal microarray. The ultrasonic scan at 29 weeks of gestation showed a fetus with abnormal posture, including persistently closed hands, flexed wrists, extended legs and knee joint contractures in addition to bilateral talipes equinovarus. In addition, there was increased thickness in the soft tissue of the forehead (7.9 mm), suggesting fetal edema (Fig. 1B). The pregnancy was terminated at 30 weeks of gestation.

Trio-based WES revealed a homozygous variant, c.1516A>C (p.Thr506Pro) in *KLHL40*, in a DNA sample from the aborted fetus. Both parents were asymptomatic heterozygous carriers (Fig. 2c).

### Patients 4 and 5

A 27-year-old nulliparous woman in her first pregnancy felt persistently reduced fetal movement. The prenatal scan at 26 weeks incidentally detected polyhydramnios. Serial ultrasound showed fetal growth retardation and progressive polyhydramnios and no obvious limb movements. The women underwent vaginal delivery at 31 weeks due to preterm premature membrane rupture resulting from polyhydramnios. A female baby weighing 1500 g was born cyanotic with no respiratory effort. The baby had generalized hypotonia, dysphagia and akinesia and required ventilation support and tube feeding. She once suffered from pleural effusion and sepsis and eventually recovered from active treatment. She was given special care, including daily physical massage and extremity fixation using bone fixation devices. At five years old, no obvious joint contractures or fractures were noted. However, she was still akinetic, with facial muscle involvement. Her eyes would open during both waking and sleeping states. She was able to slightly shake her head, move her mouth and eyes and feel emotion, but she was not able to vocalize or move her trunk, extremities and facial muscles. She can cry, maintains minimal eye contact and responds to ambient events or personal interactions. Her nerve conduction velocity was normal, and electromyography implied myopathy. Karyotype and chromosomal microarray results were normal. MLPA detected no deletion of exons 7 and 8 in *SMN1*. However, WES was not performed for the family.

The woman had another unplanned spontaneous pregnancy. Again, she experienced reduced fetal movement and progressive polyhydramnios that were strikingly similar to her previous pregnancy. The anomaly scan at 28 weeks of gestation showed a fetus with abnormal posture, including closed hands, flexed wrists, bilateral knee contractures and clubfeet. Then, the woman decided to terminate the pregnancy and undergo whole-exome sequencing.

Trio-based WES revealed a homozygous variant, c.1516A>C (p.Thr506Pro) in *KLHL40*, in a DNA sample from the aborted fetus. Both parents were asymptomatic heterozygous carriers (Fig. 2d). Next, we analyzed the DNA sample of the female patient, which had been still retained in our laboratory, and it expectedly revealed the same *KLHL40* homozygous variant.

## Discussion

**Fig. 3:**
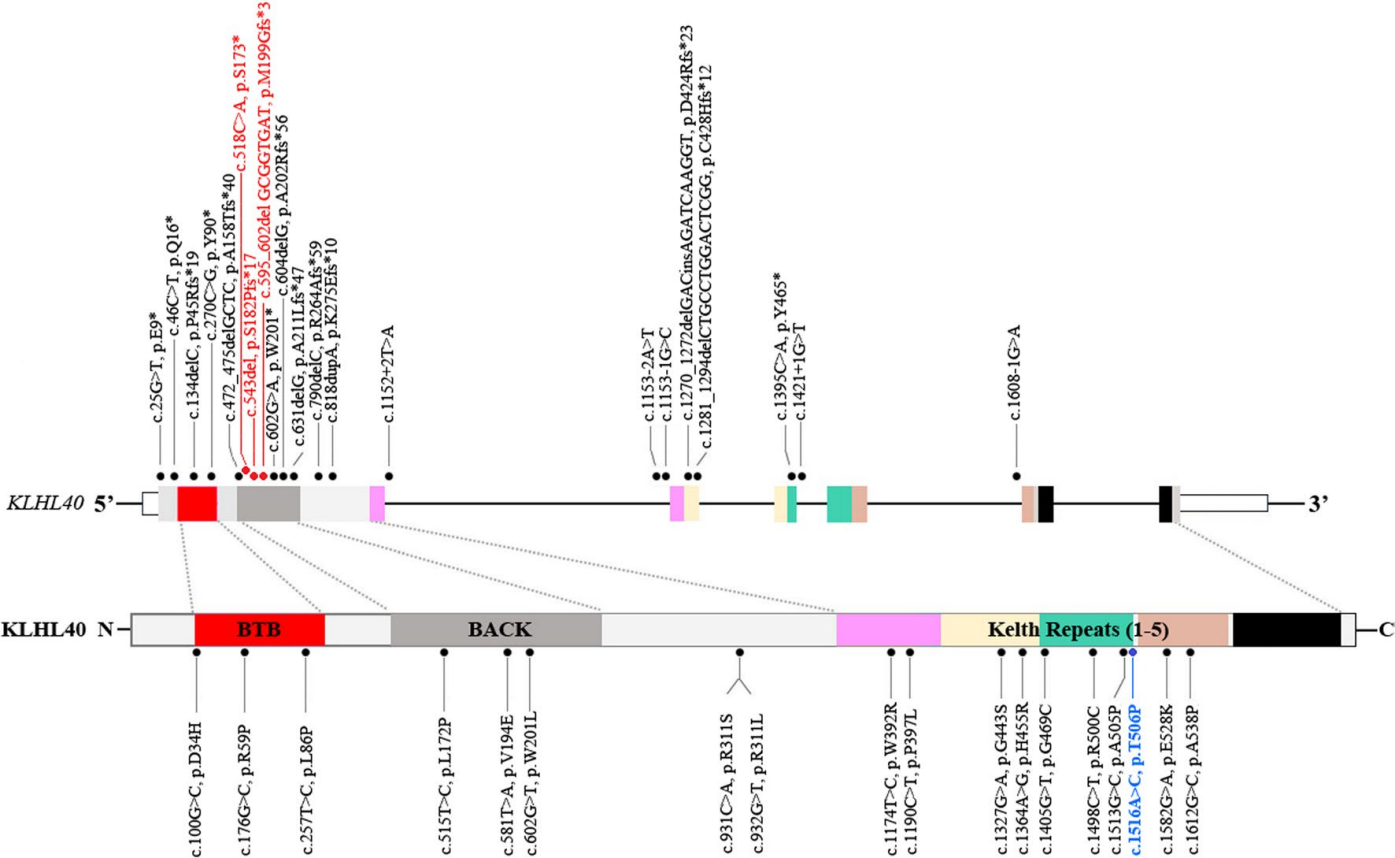
*KLHL40* variants identified to date in individuals with NEM8. Schematic presentation of the genomic structure of the *KLHL40* gene (upper) and its encoded protein, KLHL40, with the N-terminal BTB-BACK domain and 5 C-terminal kelch repeats (lower). The localization of variants and substitutions identified is depicted with dots. Black: variants reported in the literature; red: novel variants identified in this study; blue: the founder mutation in Chinese patients with NEM8

The *KLHL40* gene consists of 6 exons and encodes the 621-amino acid KLHL40 protein, which has an N-terminal BTB/BACK domain and 5 C-terminal Kelch repeats that are predicted to form a beta-propeller structure. The KLHL40 protein has the highest expression in skeletal muscle and lower expression in heart, with little to no expression in the other tissues examined [2, 11]. KLHL40 belongs to the superfamily of kelch-repeat-containing proteins that form characteristic β-propeller structures, binding substrate proteins that are involved in a wide variety of functions, such as cell migration, morphology, and protein expression. As a stabilizer of the thin filament proteins leiomodin-3 and nebulin, KLHL40 is necessary for both myogenesis and skeletal muscle maintenance [11, 12]. Homozygous or compound heterozygous variants in *KLHL40* result in NEM8, one of the most severe forms of NEM [2]. To date, only 46 individuals with NEM8 have been reported worldwide and the identified variants in *KLHL40* were scattered through all exons (Fig. 3) The main clinical manifestations include prenatal polyhydramnios (51.3%), fetal akinesia/hypokinesia (77.4%), multiple joint contractures (89.7%), spontaneous fractures (60.7%), respiratory failure (97.4%) and dysphagia (96.8%) during the neonatal period, with the average age of death being 5 months. Furthermore, most patients had facial involvement and muscle weakness. Fetal edema was an infrequent phenotype (9.7%) [2–9]. As reported, multiple joint contractures and spontaneous fractures are very common in NEM8. Surprisingly, our female patient (patient 4) never experienced joint contractures or spontaneous fractures, but her sibling (patient 5) suffered from obvious multiple joint contractures at 28 weeks fetal gestation, even though they carried the same homozygous variant, c.1516A>C (p.Thr506Pro) in *KLHL40*. This is the first report indicating that the variant c.1516A>C in *KLHL40* did not cause joint contractures or fractures, with phenotypic variability observed even within the same family. A recent report demonstrated that pyridostigmine was effective for the severe *KLHL40* phenotype [13]. To date, pyridostigmine or other neuromuscular junction-acting molecules have not been tried in the female patient. The illness of the female in this report was effectively controlled, and no sign of deterioration occurred. Her current status is similar to the locked-in state reported for a male patient with the homozygous variant c.1405G>T (p.Gly469Cys) in *KLHL40*, as reported by Kawase et al. (2015) [3]. She is the longest-lived patient with the c.1516A>C (p.Thr506Pro) variant in *KLHL40* to date. Fetal edema has been infrequently described for NEM8 [3, 5, 9], and here, we add another two cases showing a fetal edema phenotype, suggesting that fetal edema is one of the clinical features of NEM8, which needs to be explored further. Therefore, this study enriches our understanding of the clinical characteristics of NEM8.

Currently, 11 individuals from 9 unrelated Chinese families harboring 4 different *KLHL40* variants have been reported. All patients carried the c.1516A>C (p.Thr506Pro) variant in *KLHL40*, which has been suggested as a founder mutation in Chinese patients with NEM8 [5, 7–9]. Our study describes an additional five individuals from four unrelated Chinese families who all harbored the known founder mutation, with patients 3 and 4 and 5 being homozygous, further supporting that *KLHL40* c.1516A>C is a Chinese-specific founder mutation causing NEM8. Furthermore, a novel frameshift variant of *KLHL40*, c.543del (p.Ser182Profs*17), was identified in patient 1. This variant has not been reported in the medical literature, HGMD or ClinVar and was not found in the 1000 Genomes Project or the Genome Aggregation Database. The variant is predicted to truncate the protein; therefore, it was regarded as pathogenic according to ACMG/AMP guidelines. This newly identified variant further expands the *KLHL40* mutation spectrum.

Although *KLHL40*-related nemaline myopathy has rarely been reported in the Chinese population, two genetic testing centers in southern China recently proposed that the carrier frequency of *KLHL40* in the population may be underestimated and that *KLHL40* should be considered for incorporation into a carrier screening panel [8, 9]. However, this evidence is from only two genetic testing centers and is therefore limited or could be considered biased. Our genetic testing center, also located in southern China, identified five individuals from four unrelated Chinese families who were both molecularly and clinically diagnosed with NEM8. Next, we assessed the allele frequency (AF) of pathogenic variants, and we reviewed all the detected variants in *KLHL40* in our in-house database (n = 3035). Excluding variants of uncertain significance, another four pathogenic variants were revealed (Additional file 1: Table S1). The overall AF in our cohort was estimated to be nearly 0.0043; thus, the predicted incidence of NEM8 would be 4.59/10^6^. Next, to evaluate the AF of *KLHL40* in Northern China, we sought support from Chigene (Beijing) Translational Medical Research Center Co., Ltd., which is a leading company in precision medicine in Northern China that has their own carrier screening database (n = 58,540). We identified 201 individuals carrying pathogenic or likely pathogenic *KLHL40* variants (Additional file 1: Table S2). The overall AF in the database was estimated to be close to 0.0034; thus, the predicted incidence of NEM8 would be 2.95/10^6^ in Northern China. Even then, its incidence may still be underestimated for two main reasons. Some variants of uncertain significance could eventually be categorized as pathogenic. In addition, most NEM8 individuals die during the embryonic or neonatal period, so clinicians lack an understanding of this disorder, which could lead to a lack of molecular diagnoses in most cases. Thus, our study provides strong evidence suggesting that the AF of *KLHL40* has been severely underestimated historically and that this gene should be included in a carrier screening panel for the Chinese population. Given the severity and unique clinical characteristics of NEM8, clinicians should be mindful of this condition, and prenatal diagnosis is urgently needed.

## Conclusions

We reported five individuals with NEM8 from four unrelated Chinese families. The results of our study help to expand the mutation spectrum of *KLHL40* and enrich the clinical knowledge of this disorder. We strongly suggest that *KLHL40* be included in a carrier screening panel and as in first-tier testing of severe neonatal myopathy in ethnic Chinese patients, especially those with a significant family history of congenital myopathies.

## Supplementary Information

**Additional file 1.** **Table S1.** Estimated pathogenic variants in *KLHL40* in our local database on ACMG guidelines. **Table S2.** Estimated pathogenic or likely pathogenic variants in *KLHL40* in Chigene database on ACMG guidelines.

## Data Availability

The datasets used and analyzed during the current study are available from the corresponding author on reasonable request.

## Abbreviations

NEM8: Nemaline myopathy 8
WES: Whole-exome sequencing
ACMG/AMP: American College of Medical Genetics and Genomics/Association for Molecular Pathology
