# Supplementary material for: Clinical and molecular analysis of four unrelated Chinese families with pathogenic KLHL40 variants causing nemaline myopathy 8
Source: Orphanet J Rare Dis. 2022 Apr 4;17:149. doi: 10.1186/s13023-022-02306-9 (PMC8981653; doi:10.1186/s13023-022-02306-9)
Supplement: Supplementary file 1 — Additional file 1. Table S1. Estimated pathogenic variants in KLHL40 in our local database on ACMG guidelines. Table S2. Estimated pathogenic or likely pathogenic variants in KLHL40 in Chigene database on ACMG guidelines. [file 13023_2022_2306_MOESM1_ESM.docx]

**Table S1 Estimated pathogenic variants in *KLHL40* in our local database** **on ACMG guidelines**

| **Position** | **Nucleotide change** | **Amino acid change** | **No. of Het** | **Novel** |
| --- | --- | --- | --- | --- |
| chr3: 42727628 | c.518C>A | p.(S173X) | 2 | YES |
| chr3: 42727699-42727706 | c.595_602del GCGGTGAT | p.( M199Gfs*3) | 1 | YES |
| chr3:42727712 | c.602G>A | p.(W201X) | 1 | NO |
| chr3:42730455 | c.1516A>C | p.(T506P) | 1 | NO |

**Table S2 Estimated pathogenic or likely pathogenic variants in *KLHL40* in Chigene database** **on ACMG guidelines**

| **Position** | **Nucleotide change** | **Amino acid change** | **No. of Het AF in GnomAD AF local** | | |
| --- | --- | --- | --- | --- | --- |
| chr3:42727129 | c.19C>T | p.(p.Q7X) | 2 |  | 0 0.0000342 |
| chr3: 42727286 | c.176G>C | p.(R59P) | 2 |  | 0 0.0000342 |
| chr3: 42727294 | c.184delT | p.(.F62Ffs*2) | 3 |  | 0 0.0000512 |
| chr3: 42727358 | c.248delC | p.(A83Afs*116) | 7 |  | 0 0.00012 |
| chr3: 42727360 | c.250C>T | p.(Q84X) | 2 |  | 0 0.0000342 |
| chr3: 42727544_42727545 | c.434-435delGC | p.(C145Cfs*23) | 1 |  | 0 0.0000171 |
| chr3: 42727628 | c.518C>A | p.(S173X) | 3 |  | 0.0001111 0.0000512 |
| chr3: 42727653 | c. 543delC | p.(I181Ifs*18) | 4 |  | 0 0.0000683 |
| chr3: 42727655_42727656 | c.545-546delCC | p.(S182Xfs*1) | 4 |  | 0 0.0000683 |
| chr3: 42727657_42727658 | c.547-548insAG | p.(S183Kfs*17) | 4 |  | 0 0.0000683 |
| chr3:42727663 | c.553delG | p.(G185Afs*14) | 1 |  | 0 0.0000171 |
| chr3:42727681 | c.571G>T | p.(E191X) | 2 |  | 0 0.0000342 |
| chr3:442727699_42727706 | c.589-596delGCGGTGAT | p.(A197Afs*5) | 2 |  | 0 0.0000342 |
| chr3:42727712 | c.602G>A | p.(W201X) | 11 |  | 0.0001474 0.000188 |
| chr3:42727927 | c.817A>T | p.(K273X) | 3 |  | 0 0.0000512 |
| chr3:42728041 | c.931C>A | p.(R311S) | 1 |  | 0 0.0000171 |
| chr3:42728042 | c.932G>T | p.(R311L) | 8 |  | 0.00005437 0.000137 |
| chr3:42728146 | c.1036C>T | p.(Q346X) | 3 |  | 0 0.0000512 |
| chr3:42728253 | c.1143C>G | p.(Y381X) | 1 |  | 0 0.0000171 |
| chr3:42728263 | c.1152+1G>A | NA | 4 |  | 0 0.0000683 |
| chr3:42729633 | c.1153-1G>A | NA | 2 |  | 0 0.0000342 |
| chr3:42729650_42729651 | c.1169-1170insA | p.(S390Sfs*55) | 2 |  | 0 0.0000342 |
| chr3:42729671 | c.1190C>T | p.(P397L) | 1 |  | 0.00005021 0.0000171 |
| chr3:42729706 | c.1225C>T | p.(E409X) | 3 |  | 0 0.0000512 |
| chr3:42729761_42729774 | c.1280-1293delGCTGCCTGGACTCG | p.(R427Rfs*13) | 1 |  | 0 0.0000171 |
| chr3:42729765 | c.1284delC | p.(C428Cfs*39) | 2 |  | 0 0.0000342 |
| chr3:42730113 | c.1325G>A | p.(W442X) | 3 |  | 0 0.0000512 |
| chr3:42730193 | c.1405G>T | p.(G469C) | 1 |  | 0 0.0000171 |
| chr3:42730210 | c.1421+1G>A | NA | 1 |  | 0 0.0000171 |
| chr3:42730388 | c.1449T>A | p.( Y483X) | 2 |  | 0 0.0000342 |
| chr3:42730398 | c.1459A>T | p.( K487X) | 2 |  | 0 0.0000342 |
| chr3:42730437 | c.1498C>T | p.(R500C) | 1 |  | 0 0.0000171 |
| chr3:42730455 | c.1516A>C | p.(T506P) | 89 |  | 0.001303 0.0015 |
| chr3: 42730498_42730499 | c.1559_1560delAC | p.(D520Dfs*28) | 1 |  | 0 0.0000171 |
| chr3:42730521 | c.1582G>A | p.(E528K) | 15 |  | 0.0008699 0.000256 |
| chr3:42730521 | c.1582G>T | p.(E528X) | 1 |  | 0 0.0000171 |
| chr3:42730547 | c.1607+1G>A | NA | 2 |  | 0 0.0000342 |
| chr3:42732349 | c.1608-2A>G | NA | 1 |  | 0 0.0000171 |
| chr3:42732355 | c.1612G>C | p.(A538P) | 1 |  | 0 0.0000171 |
| chr3:42733373 | c.1755-1G>A | NA | 1 |  | 0 0.0000171 |
| chr3:42733377 | c.1758T>A | p.(Y586X) | 1 |  | 0 0.0000171 |
